# Popular Medicine

**Published:** 1909-10-23

**Authors:** 


### The Hospital

A Journal of

The Medical Sciences and Hospital Administration.

New Series. No. 139, Vol. VI. [No. 1211, Vol. XLVII.] SATURDAY, OCTOBER 23, 1909.

### POPULAR MEDICINE.

From time to time the lay Press becomes violently enamoured of things medical. This is especially likely to occur during epidemics of such diseases as influenza. The wise editor, well drilled to gauge (if not to create) a public taste, decides that the time is ripe for a medical lecture, and commissions a minion to obtain one. Whence? From the "well-known Harley Street physician" to be sure; that modest but voluble gentleman who is never at a loss to provide the most recent information upon all matters medical which seem fitted to make a bid for popular sympathy. But the prevalence of an epidemic disease is not by any means an essential antecedent of the medical newspaper paragraph. An infinite variety of ephemeral topics is capable of driving interviewers post haste to the kindly expert. The House of Lords Committee on the Movable Dwellings Bill, for example, has recently agitated a contemporary to the extent of a heavy headline, "Is washing good for health?" Enter forthwith the "London doctor and hygiene expert." "It is well known," says he, "that too frequent immersions in water and too lavish use of soap use up the body's natural oils, make the skin delicate, and render the whole system acutely sensitive to atmospheric changes." Not content with this authoritative pronouncement, the determined seeker after knowledge pries into the literature and secures the following guidance from a medical writer: "I find that many people imagine that it is necessary not only to plunge into hot or cold water once a day, but that they must scrub themselves all over with soap every time they do it. This is a very effective way of disinsulating the magnetic machine and depleting the individual energy. The labouring classes could not afford to take baths every day. Their exertions increase the activity of their skins, and the more active the skin is the more it cleanses itself. They could not perform their daily labours if they indulged in the luxury of a bath every day."

The above is a fair example of the common type of medical paragraph to be met with in the Press. But occasionally one meets much more pretentious efforts. For example, a provincial contemporary has lately opened its columns to two long articles upon appendicitis, from "A special correspondent." The second of these articles—we have not seen the first—is a purely medical dissertation upon the causes and symptoms of the disease. The author has solved the problem of the cause of appendicitis, and here it is: "There may not have been any gross symptoms of indigestion preceding an attack of appendicitis, and there may not have been any flagrant gluttony, but there has been error, either in the way of introducing into the stomach too much or unsuitable food (*sic*) so as to fill the intestine with semi-digested material, and if there has been simultaneous neglect to secure sufficient action of the bowels stagnation is the consequence. All this leads to overloading with decomposing material or of (*sic*) irritation of that part of the bowel to which the appendix is attached. This abnormal state leads to interference with the emptying of the appendix and the germs multiply in the pent-up secretion within the appendix and assume their virulent character, and the mischief has commenced."

It remains to notice the most imposing form of popular medicine, namely the popularly written book. This kind of literature has been a good deal to the fore lately owing to the publication of Methuen's "New Library of Medicine," a series of volumes written by respected members of our profession upon subjects a general knowledge of which is held by the editor—and, it is to be supposed, by the writers—to be of the highest importance to the public weal.

Here, then, we have the three main types of medical fare presented to the public. It would, of course, be grossly unjust to place the first and the last types in the same category for any other purpose than our present one, which is to consider the forms in which popular medicine appears, and the extent to which popular medicine is justifiable or the reverse. The subject is not a simple one. Unquestionably the bulk of the profession looks very much askance upon the popular treatment of medical subjects; partly because it is argued, and with no little justice, that the bearing of medical writings can only be appreciated justly by the trained mind, partly because the practice of writing for the people raises inevitably a suspicion of illegitimate advertising on the part of the writer. Nevertheless, it will be admitted on all hands that ignorance is the mother of an infinity of diseases, and that the education of the multitude in matters of hygiene—which is after all but preventive medicine—is a condition precedent to success in the campaign against disease. We may say at once that in our judgment the man who can write popularly upon medical subjects and at the same time with propriety and benefit to his readers, is very rare indeed. We may dismiss as entirely unworthy of notice the windy futilities of the "Harley Street physician." The writer upon appendicitis, and his likes, seem to us to merit unqualified condemnation, for nothing but harm can ensue from suggesting to people that whenever they get a twinge of colic they should apprehend appendicitis. Those who read such articles will be either the idly curious, who will hope for something sensational in them, or the anxious and valetudinarian whose already sufficient alarms are likely to be intensified by the reading. Such writing is at the best unnecessary, and at the worst extremely pernicious. As for serious and reputable books of popular medicine, it may be said that much turns upon the subject chosen, but even more upon the handling of it. As a matter of experience we should say that the good effected by these books is small, out of all proportion to the labour expended upon their compilation. We do not know whether it is that the writers yield to the insistence of publishers and are tempted to spread their net as widely as possible, or whether there be some other explanation, but a perusal of such books as we are considering leaves one with the impression that the author has had one eye on the profession and the other on the public. In consequence the result, as a general rule, is a hotch-potch of incompatibles, of technicalities which must certainly be Dutch to the layman, and of elementary and platitudinous observations extremely forbidding to the professional reader. We believe there is room for genuinely popular books on many matters connected with health and disease, but it is an almost hopeless task to appeal simultaneously to the profession and the public.

